# The role of BCL9 genetic variation as a biomarker for hepatitis C-related hepatocellular carcinoma in Egyptian patients

**DOI:** 10.1186/s43141-021-00282-4

**Published:** 2022-01-03

**Authors:** Eman Abd El Razek Abbas, Ahmed Barakat Barakat, Mohamed Hassany, Samar Samir Youssef

**Affiliations:** 1 Microbial Biotechnology Department, National Research Centre, 33 El-Buhouth St., Dokki, Giza, Cairo 12622 Egypt; 2 Microbiology Department, Faculty of Science, Ain-Shams University, Cairo, Egypt; 3 Tropical Medicine Department, National Hepatology and Tropical Medicine Research Institute, Cairo, Egypt

**Keywords:** Hepatocellular carcinoma, BCL9, Copy number variation, Circulating free DNA, Biomarker

## Abstract

**Background:**

Hepatocellular carcinoma (HCC) is considered one of the most common cancers related to mortality around the world, and susceptibility is related with genetic, lifestyle, and environmental factors. Copy number variation of the Bcell CLL/lymphoma 9 (BCL9) gene is a type of structural variation which can influence gene expression and can be related with specific phenotypes and diseases and has a role in hepatocarcinogenesis. Our aims were to assess the copy number variation (CNV) in the BCL9 gene and explore its role in HCV-related HCC Egyptian patients. A total of 50 HCV-related HCC patients were enrolled in the study (including 25 early HCC and 25 late HCC cases); the copy number of the BCL9 gene was detected using quantitative polymerase reaction.

**Results:**

There was a highly statistically significant difference between the two groups (early and late HCC patients) in gender, bilharziasis, performance status, child score class, child grade, focal lesion size, portal vein, and ascites. CNV was detected and represented by the gain in the BCL9 gene in 14% of patients, and all of them were males. Also, it was noticed that the ratio of gain in BCL9 copy number in late individuals was about 1.5 times than that in early HCC individuals. Moreover, our results showed that the distribution of performance status > 1, average and enlarged liver, focal lesion size, thrombosed portal vein, and AFP was higher in patients with BCL9 copy number gain.

**Conclusion:**

We detected about 14% gain in BCL9 copy number in Egyptian HCC patients. But the variation in copy number of the BCL9 gene did not affect HCC development in our patients’ cohort.

## Background

Hepatocellular carcinoma (HCC) is one of the most universal problems worldwide, it is the 6th most common cancer and the 4th cause of cancer-related death all over the world. There is variation in the predominance of HCC in the epidemiological data from one place to another [1, 2]. In Egypt, HCC ranks the 4th common cancer, and it is considered the most reason for mortality- and morbidity-associated cancer [3, 4].

There are many risk factors in HCC development, which include [1] environmental factors such as hepatitis B virus (HBV) and hepatitis C virus (HCV) infection, chemical compounds, alcohol, and smoking [2]; host-related risk factors such as gender, ethnicity, obesity, autoimmune hepatitis, diabetes, and non-alcoholic fatty liver disease (NAFLD) [3]; genetic-related factors such as the history of HCC, aflatoxins, and genetic alterations [5]. Both HBV and HCV increase HCC risk by 20-fold [6–8]. In Egypt, HCV is the most critical risk factor in liver cancer including HCC; about 21.4% of HCC individuals have a family history of HCC [9, 10].

Although the survival rate is approximately 5 years, this rate is estimated to be 18%, and the median survival is only 7 months, if untreated [11–14]. HCC patients are often diagnosed at the advanced phase when treatment is restricted to the systemic therapy and multi-kinase inhibitors which are combined with side effects [15, 16]. So, we are in a critical need for characterization of the molecular mechanistic targets in HCC and tumor-specific signaling pathways, which are still a major goal of HCC research, to diagnose and treat it at the early stage.

Also, it was shown that human genetic variations such as genetic mutation, single nucleotide polymorphism (SNP), copy number variation (CNV) affect the susceptibility to liver cancer and carcinogenesis [17, 18].

Copy number variations have been discovered as intra-species alterations, which occurs as a repeating of a sequence of nucleotides in tandem multiple times in an individual’s genome, including insertion (gain) or deletion (loss) of genetic material with a highly significant biological role; they range in the human genome from 10 kb to 1 Mb in size and cover about 12% of the human genome [19, 20]. These CNVs can change the gene structure and gene expression and lead to significant phenotypic variation [21]. Many studies have reported a critical role for CNVs in important molecular mechanisms of pathogenesis in autoimmune diseases and infectious diseases [22–25].

The previous studies identified that several genes involved in many diseases exhibit CNV such as CYP2D6, MMP-6, BCL9, and BCL9L; these CNVs can be considered as prognostic biomarkers for many diseases [26–31]. CN gains on 1q, 8q, and 20q and losses on 1p, 8p, 4q, 13q, 16q, and 17p have been previously recognized in HCC [32–34].

The human Bcell CLL/lymphoma 9 (BCL9) gene, located at chromosome 1q21, was discovered as an overexpressed gene in a precursor B cell acute lymphoblastic leukemia cell line with t(1;14)(q21;q32) translocation [35]. It was found that BCL9 is an essential co-activator in the Wnt/β-catenin signaling pathway, enhances β-catenin-mediated transcription activity, and increases cell proliferation, invasion, migration, and metastatic potential of tumor cell [36, 37].

BCL9 has an oncogenic function as it is overexpressed in many solid tumors including HCC and colorectal cancer; this is combined with a poor prognosis of HCC cases [38–40]. Previous studies have examined the role of BCL9 in cancer, including HCC [27, 30, 41]. A copy number gain on 1q is frequently observed in HCC; the BCL9 regional chromosomal gain represents a primary mechanism in the activation of proto-oncogenes during HCC progression [27, 28, 33]. The prognostic significance of BCL9 in HCC remains uncertain. However, in hepatocellular tumors, the deletion of BCL9 increased survival rates, reduced liver size and cell proliferation, decreased expression of Wnt target genes, and inhibited the HCC migration and invasion [30, 41, 42]. So, BCL9 can be the center stage in the HCC treatment.

In the studies of HCC, the role of BCL9 expression has not been characterized especially in Egypt. Therefore, in the present study, we evaluated BCL9 CNV by qPCR in order to elucidate the prognostic significance of BCL9 in HCC-infected patients with clinopathological characteristics.

## Methods

### Study population

A total of 50 patients were enrolled in the present study including (25 HCC-infected patients in the early stage and 25 HCC infected patients in the last stage). All cases were gathered from the National Hepatology and Tropical Medicine Research Institute, Cairo, Egypt, between December 2019 and April 2020. Subjects who had co-infection with HBV, HIV, EBV, CMV, bilharziasis, diabetes mellitus, autoimmune diseases, and other cancers and also had a history of alcohol consumption were excluded from the study.

This study was approved by the Ethics committee of the Medical Research, registration number (17122), National Research Centre, Dokki, Cairo, Egypt, and all participants signed the patient’s concept form for this research. Fibrosis was assessed by FIB4 score. Some patients had a biopsy report assessed by Metavir. This study was approved by the local research ethics committee, and written informed consent was obtained from all participants in this study.

### Blood collection and plasma separation

Collect about 10 ml peripheral blood in EDTA tubes, then centrifuge the tubes immediately at 3000 rpm for 10 min, at room temperature, then at 14,000 rpm for 15 min at 4 °C. Finally, transfer the supernatant plasma into 2-ml tubes and preserve them at once at − 80°C until further use.

### Circulating-free DNA extraction

Circulating-free DNA (cfDNA) was isolated from the frozen plasma using the QIAamp Circulating Nucleic Acid Kit (Qiagen#55114) following the manufacturer’s instructions and store at − 20 °C for genetic determination. The concentration of the purified cfDNA was determined by NanoDrop™ One/OneC Microvolume UV-Vis Spectrophotometer (Catalog number: 701-058108, Thermo Fisher Scientific, Invitrogen).

### Measurement of BCL9 copy number

The copy number variations were determined by using Real-Time Quantitative PCR [43, 44]. The copy number of the BCL9 gene was measured in each cfDNA sample using the Mx3000p comparative quantitative PCR system (Agilent Technologies, Germany) and TaqMan copy number assay. TaqMan CN assay is a duplex experiment using The FAM™ dye-labeled TaqMan® Copy Number Target Assay for BCL9 (Assay id:Hs00964453_-_cn, Cat. No. 4400291, Applied Biosystem, USA), with the VIC® dye-labeled TaqMan® Copy Number Reference Assay for RNase P (RPPH1) (Assay id:4403326, Applied Biosystem, USA) in the same well to quantity small fold changes. The quantitative polymerase chain reaction (qPCR) was carried out in 20 μl volume using 10 μl (2×) TaqMan genotyping master mix (Applied Biosystem, USA), 1 μl (20×) TaqMan copy number reference assay RNase P (RPPH1) as an internal control (Assay id:4403326, Applied Biosystem, USA), 1 μl (20×) Target BCL9 copy number assay (Assay id:Hs00964453_-_cn, Cat. No. 4400291, Applied Biosystem, USA), 4 μl cfDNA sample (5 ng/μl concentration), and complete the reaction volume with DNAse-free water (Promega, USA). TaqMan target CN assay and TaqMan® Copy Number Reference Assay are in the same well to quantity small fold changes. All assays were performed in triplicates of each sample and a calibrator sample (healthy sample) and negative control (with water instead of cfDNA sample) in 96-well PCR plate for amplification, using the following program: 95 °C for 10 min as the initial melting, followed by 40 cycles at 95 °C for 15 s and 60 °C for 1 min.

The copy number was calculated by the ΔΔCt method [45] to obtain the relative quantity (RQ) then multiply it by the copy number of the calibrator, which in this case are 2.

### Statistical analysis

The statistical analysis was performed using the Social Sciences (SPSS) version 26 (IBM Corp., Armonk, NY, USA). Data were summarized using a median and interquartile range in normally quantitative data, using mean ± standard deviation in non-normally distributed data, and using frequency (count) and relative frequency (percentage) for categorical data. Comparison between the distributions of categorical variables was performed using the chi-square (*χ*^2^) method, and comparisons between quantitative variables were done using the non-parametric Kruskal-Wallis test. Moreover, variables were described as odds ratio (OR) with 95% confidence interval (95% CI) by using crosstabs and binary logistic regression analysis. All *P*-values were 2-sided, and *P*-values less than 0.05 were considered as statistically significant and less than 0.01 were considered highly significant.

## Results

### Demographic data and clinical investigation of the study population and Association of HCC characteristics and baseline parameters with HCC development

The demographic, biochemical, virological, and histopathological data of all 50 HCV-related HCC-infected patients are summarized in Table 1. Patients have a mean age of 63.14 with 78% of males, about 84% of patients have higher AFP, and all patients were cirrhotic. Fifty percent of patients were at the early stage, and the other 50% were at the late stage, the majority of patients have child score class B, patent portal vein, and no ascites.

**Table 1:** Demographic and clinical parameters of the early versus late HCC patients

Parameters			HCC patients, ***N*** = 50	HCC stage		OR (95% CI)	Chi-square	***P***-value
				Early stage, ***N*** = 25	Late stage, ***N*** = 25			
**Demographic data**	Age (mean ± SD), years		63.14 ± 7.89	64.00 ± 8.54	62.28 ± 7.25	0.972 (0.903–1.045)	1.001	0.317
	Gender, male, no. (%)		39 (78.0%)	15 (60.0%)	24 (96.0%)	0.063 (0.007–0.539)	9.441	0.002**
	BMI (mean ± SD), kg/m^2^		25.75 ± 3.58	26.15 ± 3.96	25.36 ± 3.20	0.938 (0.799–1.102)	0.475	0.491
**Laboratory investigation**	Anti-bilharzial treatment	Yes	15 (30.0%)	4 (16.0%)	11 (44.0%)	0.242 (0.064–0.916)	4.667	0.031*
		No	35 (75.0%)	21 (84.0%)	14 (56.0%)			
	Hb (mean ± SD), g/dl		12.76 ± 1.83	13.08 ± 1.15	12.46 ± 2.30	0.816 (0.585–1.138)	2.724	0.099
	TLC (mean ± SD), 103/mm^3^		5.54 ± 2.33	5.17 ± 1.77	5.91 ± 2.76	1.155 (0.896–1.490)	0.462	0.497
	PLT (mean ± SD), 103/mm^3^		140.54 ± 68.14	132.24 ± 61.83	148.84 ± 74.25	1.004 (0.995–1.012)	0.502	0.479
	AST (mean ± SD), U/l		44.0 (34.0–77.0)	43.0 (36.0–64.0)	52.0 (34.0–82.0)	1.004 (0.986–1.022)	0.115	0.734
	ALT (mean ± SD), U/l		39.0 (30.0–59.0)	38.0 (30.0–54.0)	48.0 (32.0–60.0)	1.007 (0.988–1.026)	0.780	0.377
	Bil.T (mean ± SD), mg/dl		1.0 (0.8–1.4)	1.0 (0.7–1.3)	1.0 (0.9–1.6)	1.426 (0.521–3.907)	0.870	0.351
	AFP, no. (%)	< 4.5	8 (16.0%)	3 (12.0%)	5 (20%)	1.000 (0.998–1.001)	0.122	0.702
		≥ 4.5	42 (84.0%)	22 (88.0%)	20 (80%)			
	Creat (mean ± SD), mg/dl		1.01 ± 0.30	0.86 ± 0.19	1.15 ± 0.32	0.964 (0.623–1.489)	10.960	0.001**
**HCC characteristics**	BCLC	0	10 (20.0%)	10 (40.0%)	0 (0.0%)	–	50.000	0.001**
		A	15 (30.0%)	15 (60.0%)	0 (0.0%)			
		C	21 (42.0%)	0 (0.0%)	21 (84.0%)			
		D	4 (8.0%)	0 (0.0%)	4 (16.0%)			
	F.H of HCC	Yes	8 (16.0%)	4 (16.0%)	4 (16.0%)	1.000 (0.220–4.536)	0.000	1.000
		No	42 (84.0%)	21 (84.0%)	21 (28.0%)			
	Performance status	0	32 (64.0%)	25 (100.0%)	7 (28.0%)	0.242 (0.1333–0.443)	25.758	0.001**
		< 1	1 (2.0%)	0 (0.0%)	1 (4.00%)			
		> 1	17 (34.0%)	0 (0.0%)	17 (68.0.0%)			
	Liver size	Average	24 (48.0%)	14 (56.0%)	10 (40.0%)	–	2.087	0.352
		Enlarged	23 (46.0%)	9 (36.0%)	14 (56.0%)			
		Shrunken	3 (6.0%)	2 (8.0%)	1 (4.0%)			
	Child score	A	36 (72.0%)	24 (96.0%)	12 (48.0%)	26.000 (3.032–222.928)	14.286	0.001**
		B	14 (28.0%)	1 (4.0%)	13 (52.0%)			
	Child grade		5.92 ± 0.57	5.36 ± 0.57	6.48 ± 1.19	3.817 (1.691–8.615)	12.585	< 0.001**
	Number of focal lesions	Single	30 (60.0%)	14 (56.0%)	16 (64.0%)	0.716 (0.230–2.230)	3.406	0.182
		Multiple	20 (40.0%)	11 (44.0%)	9 (36.0%)			
	Focal lesion size		3.45 (2.00–5.90)	2.24 ± 0.85	6.33 ± 3.64	3.103 (1.569–6.135)	21.677	< 0.001**
	P.V.	Patent	42 (84.0%)	25 (100.0%)	17 (66.0%)	0.405 (0.280–0.584)	9.524	0.002**
		Thrombosed	8 (16.0%)	0 (0.0%)	8 (32.0%)			
	Ascites	Yes	11 (22.0%)	2 (8.0%)	9 (36.0%)	0.155 (0.029–0.813)	5.711	0.017*
		No	39 (78.0%)	23 (92.0%)	16 (64.0%)			
	Cirrhosis, no. (%)		50 (100%)	25 (50%)	25 (50%)			

The data were analyzed by the non-parametric Kruskal-Wallis and chi-square tests

*BMI* body mass index, *HB* hemoglobin, *TLC* total leukocyte count, *PLT* platelet, *AST* aspartate aminotransferase, *ALT* alanine transferase, *Bili T* bilirubin total, *AFP* alpha-fetoprotein, *Creat* creatinine, *BCLC* Barcelona Clinic Liver Cancer, *F.H* family history, *P.V* portal vein, *OR* odds ratio, *CI* confidence interval

*P-value ≤ 0.05 significant

**P-value ≤ 0.01 highly significant

Univariate analysis of our data in Table 1 showed that there was a highly statistically significant difference between the two groups (early and late HCC patients) in gender, BCLC, bilharziasis, performance status, child score class, child grade, focal lesion size, portal vein and ascites, and creatinine. Ninety-six percent of late HCC patients were males, about half of late HCC patients (56%) had enlarged liver in size; on the contrary, half of early HCC patients (56%) had an average liver. Also, it was found that all early HCC patients had no thrombosed portal vein (0.00%) and 92% of them had no ascites.

### Variation of copy number of BCL9 in male versus female HCC patients

**Fig. 1:**
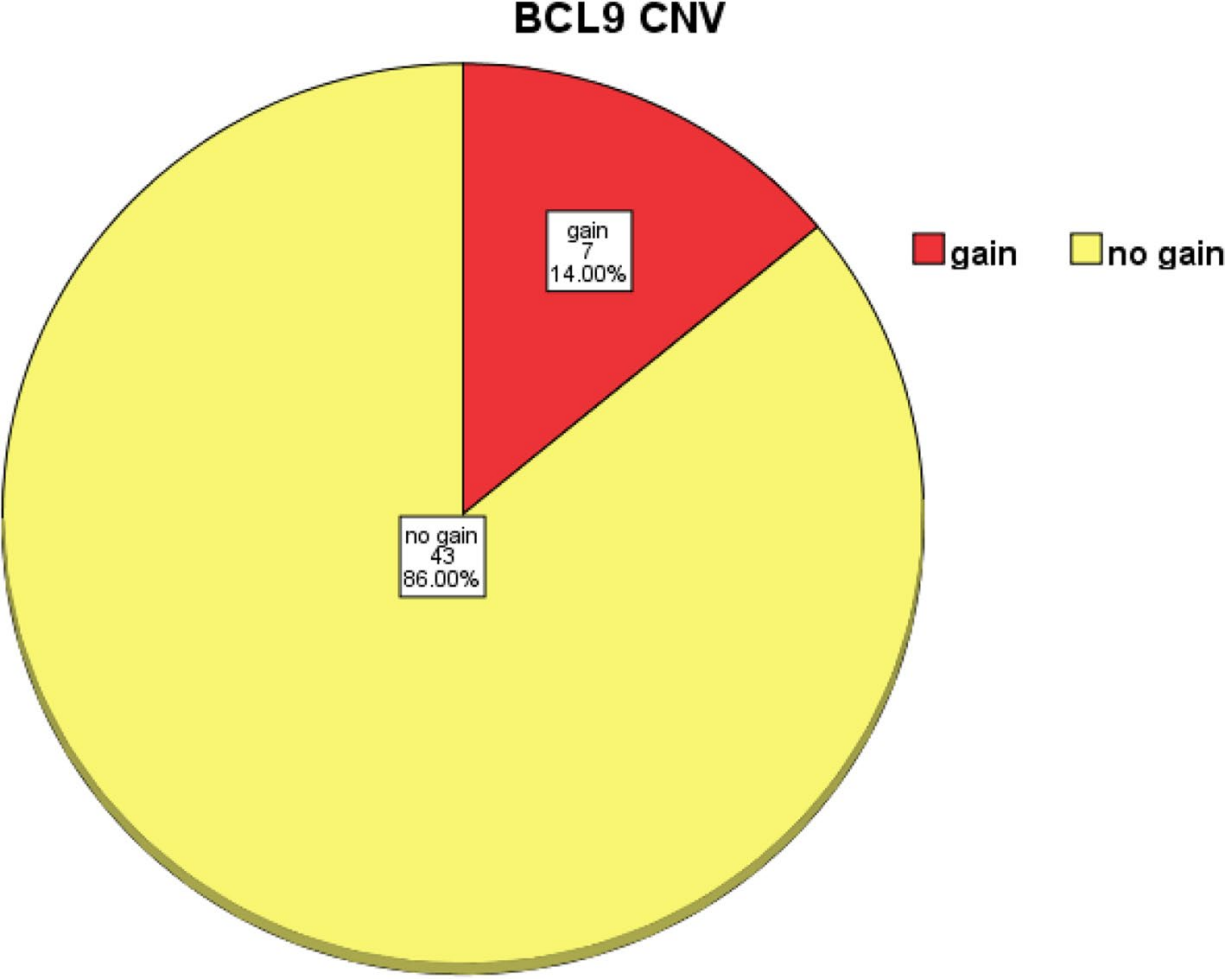
Pie chart of distribution of BCL9 CNV in HCC patients. The figure shows the copy number distribution of the BCL9 gene. Seven HCC patients (14%) had gain in BCL9 CN, and 43 patients (86%) had no gain in BCL9 CN. Gain: CN gain, no. = 7, % = 14; no gain: no gain in CN, no. = 43, % = 86

Among our studied population of HCC patients, CNV was detected and represented by the gain in BCL9 gene in 14% of patients and all of them were males as in Fig. 1, and CNV gain was not documented in female HCC patients. CNV frequencies in the BCL9 gene are outlined in Table 2.

**Table 2:** Distribution of CNV in HCC patients

CNV	All HCC patients, ***N*** = 50	Male patients, ***n*** = 39	Female patients, ***n*** = 11	***P***-value
**Gain**	7 (14%)	7 (17.95%)	0 (0%)	0.324
**No gain**	43 (86%)	32 (82.05%)	11 (100%)	

*CNV* copy number variation

### Correlation of CNV in the BCL9 gene in HCC patients with HCC development

CNV in the BCL9 gene showed differential distribution within early and late HCC patients as shown in Table 3. The distribution of gain within early and late HCC individuals was 12% and 16%, respectively, so it was noticed that the ratio of gain in BCL9 copy number in late individuals was about 1.5 times than that in early HCC individuals.

**Table 3:** BCL9 CN gain in HCC patients according to early and late HCC stages

CNV	Early HCC patients, ***N*** = 25	Late HCC patients, ***n*** = 25	***P***-value
**Gain**	3 (12.0%)	4 (16.0%)	1.00
**No gain**	22 (88.0%)	21 (84.0%)	

### Correlation between variation in the BCL9 gene CN in male patients and the clinical characteristics of HCC patients

The differential alteration was seen in clinical and HCC characteristics parameters according to CNV in the BCL9 gene as outlined in Table 4. This difference did not reach a significant association in all studied parameters; our results showed that 57% of those carrying gain in CNV were at an advanced stage of HCC (BCLC stage C). The frequency of family history of HCC in males within CNV gain HCC patients was few (14.29%).

**Table 4:** BCL9 CN gain according to male gender and clinical and HCC characteristics parameters

Parameters		Total male patients, ***N*** = 39	CNV in male patients		***P***-value
			Gain, ***N*** = 7	No gain, ***N*** = 32	
Age (mean ± SD), years		62.64 ± 8.34	64.00 ± 5.69	62.34 ± 8.86	0.761
BMI (mean ± SD), kg/m^2^		25.0 ± 3.10	23.57 ± 3.02	25.32 ± 3.08	0.212
Hb (mean ± SD), g/dl		12.73 ± 1.97	12.90 ± 2.04	12.69 ± 1.99	0.707
TLC (mean ± SD), 103/mm^3^		5.60 ± 2.52	5.08 ± 1.75	5.72 ± 2.67	0.654
PLT (mean ± SD), 103/mm^3^		141.82 ± 69.38	143.71 ± 64.76	141.41 ± 71.33	0.629
AST (mean ± SD), U/l		55.15 ± 33.77	66.86 ± 42.98	52.59 ± 31.67	0.484
ALT (mean ± SD), U/l		49.26 ± 35.31	55.0 ± 26.64	48.00 ± 37.1	0.287
Bil.T (mean ± SD), mg/dl		1.15 ± 0.52	1.07 ± 0.50	1.17 ± 0.53	0.603
AFP, no. (%)	< 4.5	5 (12.82%)	1 (14.29%)	4 (12.50%)	1.000
	≥ 4.5	34 (87.18%)	6 (85.71%)	28 (87.5%)	
BCLC	0	6 (15.38%)	1 (14.29%)	5 (15.6%)	0.929
	A	9 (23.08%)	2 (28.57%)	7 (21.87%)	0.653
	C	20 (51.28%)	4 (57.14%)	16 (50.0%)	0.732
	D	4 (10.26%)	0 (0.0%)	4 (12.5%)	0.323
F.H of HCC	Yes	6 (15.38%)	1 (14.29%)	5 (15.63%)	1.00
	No	33 (84.62%)	6 (85.71%)	27 (84.37%)	
Performance status	0	22 (56.41%)	3 (42.86%)	19 (59.38%)	0.426
	< 1	1 (2.56%)	0 (0.0%)	1 (3.12%)	0.636
	> 1	16 (41.03%)	4 (57.14%)	12 (37.5%)	0.339
Liver size	Average	18 (46.15%)	4 (57.14%)	14 (43.75%)	0.520
	Enlarged	18 (46.15%)	3 (42.86%)	15 (46.88%)	0.847
	Shrunken	3 (7.70%)	0 (0.0%)	3 (9.37%)	0.399
Child score	A	26 (66.67%)	4 (57.14%)	22 (68.75%)	0.666
	B	13 (33.33%)	3 (42.86%)	10 (31.25%)	
Child grade		6.08 ± 1.13	6.0 ± 1.0	6.09 ± 1.17	0.957
No. of focal lesions	Single	23 (58.97%)	5 (71.43%)	18 (56.25%)	1.00
	Multiple	16 (41.03%)	2 (28.57%)	14 (43.75%)	
Focal lesion size		4.83 ± 3.46	5.86 ± 4.85	4.60 ± 3.14	0.707
P.V.	Patent	7 (17.95%)	2 (28.57%)	5 (15.63%)	0.588
	Thrombosed	32 (82.05%)	5 (71.43%)	27 (84.37%)	
Ascites	Yes	9 (23.08%)	2 (28.57%)	7 (21.87%)	0.653
	No	30 (76.92%)	5 (71.43%)	25 (78.13%)	

The data were analyzed by the non-parametric Kruskal-Wallis and chi-square tests.

*P-value ≤ 0.05 significant

**P-value ≤ 0.01 highly significant

Also, the frequencies of performance status > 1, average and enlarged liver, focal lesion size, and thrombosed portal vein were higher in male patients having BCL9 CN gain.

Although there was no statistical significance between gain in CN of BCL9 and AFP, the majority of patients (about 85%) who have CNV gain have higher AFP values. Additionally, AST and ALT values were higher in the CNV gain group.

## Discussion

HCC is considered the most common primary liver cancer, it accounts for about 90% of cases, which develops mostly in the setting of chronic liver disease [3, 46].

There is gender variation of HCC incidence; as in this current study, 78% of HCC cases were males, and this agrees with the previous studies which showed that HCC predominantly is more common in males [11, 47]. The strongest risk factor for HCC development is cirrhosis, about 80% of HCCs develop in the cirrhotic liver [48–50]. Here, we found that all patients have a cirrhotic liver.

The prognosis of patients with this tumor remains poor [50]. Progression pattern may be a key prognostic parameter and important pattern for the fate of HCC, and the performance of histological biomarkers with clinical data has shown promise for surveillance and early diagnosis [1]. In this study, our results showed a highly significant correlation between stages of HCC groups (early and late HCC) and gender, bilharziasis, performance status, child score and grade, focal lesion size, portal vein, and ascites. Our results showed that most of our late HCC patients had enlarged liver, while our early HCC patients group lacks thrombosed portal vein and ascites, this is consistent with previous reports [51–55]. Also, these results are in line with the results of Hyeon et al., who confirmed that tumor stage, tumor recurrence, and survival were significantly associated with larger tumor size, higher Edmondson grade, microvascular invasion, major portal vein, higher BCLC grade, higher AFP, liver cirrhosis, and viral etiology [27].

Also, it is known that HCC is a heterogenous pathology, involving many somatic genetic mutations; these genetic alterations and abnormal activation of specific pathology are responsible for HCC development, tumor differentiation, and related clinical outcomes [6, 7, 56]. Copy number (CN) in genomic DNA is one of the most observed genetic alterations and chromosomal instability in HCC and can be used as an indicator of major disease [31–34, 57].

A gain in 1q chromosome is one of the most frequently detected chromosomal alterations in HCC and has been suggested as an early genomic event in the HCC development [58]. BCL9 is considered one of the important oncogenes located at 1q21.1 and encodes B cell CLL/lymphoma 9 which is considered as co-activator of β-catenin-mediated transcription in the Wnt/β-catenin signaling, and its upregulation plays a vital role in certain cancer development and progression, including colorectal cancer and HCC [37, 38]. On the other hand, it inhibits tumor growth and increases the survival outcome of multiple myeloma and colon cancer through blocking the Wnt/β-catenin pathway [30, 41]. Wang et al. credentialed BCL9 as a candidate driver gene as it plays a significant role in HCC growth and survival and may serve as a potential therapeutic target for the disease treatment, as it found a significant correlation between BCL9 somatic copy number and gene expression in primary HCCs [59]. Also, other studies have examined the role of BCL9 in cancer, including HCC [27, 30, 41, 42].

In this study, we focused on studying the alteration in CN of BCL9 in Egyptian HCC patients as a crucial risk factor in HCC development. To the best of our knowledge, this is the first study to use CN analysis of BCL9 to explore the relation between CNV and HCC development, and the role of BCL9 in HCC has not been reported previously in Egypt.

Our results showed variation in BCl9 CN towards gain which was detected in 14% of our patients; this is in agreement with Wang et al.’s study which found that BCL9 was highly amplified in 8.7% of the HCC cohort [59]. Also, we found that all of CN gain was in male patients; this information has not been discussed before and may be one of the reasons why HCC is more common in males, but this needs more research to confirm this theory.

We further noticed that the ratio of CN gain in HCC individuals at the advanced stage was higher than the early stage, and this may reflect the extent of the BCL9 CN prognostic role in HCC development. This matches with other studies which found that somatic CN of BCL9 gene was associated with advanced HCC tumor stage [30, 59, 60].

Here, our results indicated that BCL9 CN gain was associated with the higher performance status, enlarged liver size, higher focal lesion size, and thrombosed portal vein; although this association did not reach the significance, this may lead to that BCL9 CNV might have a role in HCC development, and there was not any significant association between this CN gain and the other parameters. This is in line with the other studies which suggest the involvement of BCL9 in the HCC pathogenesis and prognosis [27, 30, 61].

An important point was discussed in our results; AFP status was higher in the majority of the BCL9 CN gain group, as AFP is the most common marker for HCC diagnosis [62]; therefore, we speculate that there is a vital role of AFP to be taken into consideration as an influencer with BCL9 CNV in HCC patients, but further work is needed to verify our hypothesis.

## Conclusion

This study is the first to discuss BCL9 CNV in HCC Egyptian patients; it recorded that there is gain in BCL9 CNV in 14% of HCC patients and also showed that CN gains existed only in male patients. Our finding indicated that CNV of BCL9 did not affect HCC development in our patient cohort, but it was found that gender, bilharziasis, performance status, child score class, child grade, focal lesion size, portal vein, and ascites have correlated with HCC development and prognosis.

### Limitations

One of the main limitations in our work is the lack of healthy individuals to compare BCL9 CNV in HCC patients and healthy individuals. Second is the small sample number enrolled in the current study of Egyptian HCC patients. So, future studies should focus on a large number of HCC patients to confirm our results.

## Data Availability

All data generated or analyzed during this study are included in this article.

## Abbreviations

HCC: Hepatocellular carcinoma
CN: Copy number
CNV: Copy number variation
BCL9: Bcell CLL/lymphoma 9
HCV: Hepatitis C virus
HBV: Hepatitis B virus
NAFLD: Non-alcoholic fatty liver disease
SNP: Single nucleotide polymorphism
CYP2D6: Cytochrome P450 family 2 subfamily D member 6
MMP-6: Matrix metalloproteinase 6
BCL9L: B cell CLL/lymphoma 9 like
HIV: Human immunodeficiency virus
EBV: Epstein-Barr virus
CMV: Cytomegalovirus
EDTA: Ethylenediaminetetraacetic acid
cfDNA: Circulating-free DNA
QPCR: Quantitative polymerase chain reaction
RQ: Relative quantity
*χ*^2^: Chi-square
OR: Odds ratio
CI: Confidence interval
SD: Standard deviation
BCLC: Barcelona Clinic Liver Cancer
